# Evaluating the effect of two different training interventions on improving vaginal twin birth rates and provider confidence and knowledge levels: A pre‐ and post‐intervention study

**DOI:** 10.1002/ijgo.70947

**Published:** 2026-03-07

**Authors:** Claudio Celentano, Barbara Matarrelli, Maurizio Rosati, Martina Mercaldi, Claudio Meloni, Cecilia Molino, Aurora Padelli, Fiorella Conti, Federico Prefumo, Rixa Freeze

**Affiliations:** ^1^ Department of Obstetrics and Gynecology Santo Spirito Hospital, Affiliated University G. d'Annunzio Chieti‐Pescara Pescara Italy; ^2^ EASC, Santo Spirito Hospital Pescara Italy; ^3^ Breech Without Borders Crawfordsville Indiana USA; ^4^ Department of Obstetrics and Gynecology SS. Annunziata Hospital, University G. d'Annunzio Chieti‐Pescara Chieti Italy; ^5^ Department of Obstetrics and Gynecology S. Giovanni di Dio Hospital Florence Italy; ^6^ Department of Obstetrics and Gynecology Careggi University Hospital Florence Italy; ^7^ Department of Maternal‐Infancy Careggi University Hospital Florence Italy; ^8^ Department of Obstetrics and Gynecology IRCCS Istituto Giannina Gaslini Genoa Italy

**Keywords:** obstetric emergencies, planned cesarean section, planned vaginal delivery, simulation in obstetrics, twins

## Abstract

**Objective:**

Despite the increasing trend in cesarean section rates in multiple pregnancies, vaginal delivery in twin pregnancies appears both possible and safe.

**Methods:**

This retrospective multicenter study compared outcomes before and after the implementation of a vaginal twin intervention in two Italian hospitals. In January 2021 in Pescara and in July 2022 in Florence, physicians with expertise in twin and breech vaginal birth started working in each center, and clinicians and midwives started quarterly training in these skills. The study period was divided into two equal time intervals and lasted 8 years in Pescara and 5 years in Florence. Providers were also surveyed about how the intervention affected their confidence, skill, and knowledge levels. Diamniotic pregnancies with two viable twins, ≥34 weeks of gestation, and cephalic presentation of the first twin were included. A total of 278 women were evaluated, split into pre‐intervention (*n* = 131) and post‐intervention (*n* = 147) periods.

**Results:**

Vaginal birth rates increased from 9.2% to 40.8% overall, with no negative effect on maternal or neonatal outcomes. Participating providers felt more skilled, knowledgeable, and confident and agreed that the training improved vaginal birth rates as well as their vaginal breech skills.

**Conclusion:**

In settings with a high background cesarean section rate, a rapid increase in the vaginal birth rate of twins is possible without adversely affecting maternal or neonatal outcomes, via either staff‐wide training or specialist teams. Simulation and skills training can improve provider confidence, knowledge, and skill levels as well as impact clinical practice in the labor ward.

**Abbreviations**
ACOG: American College of Obstetricians and Gynecologists
ART: assisted reproductive technologies
CS: cesarean section
MCDA: mono‐chorionic di‐amniotic
NICU: neonatal intensive care unit
PPH: post partum hemorrhage
VB: vaginal birth

## 1 INTRODUCTION

Twin pregnancies account for approximately 2%–4% of all births worldwide,^1^ but this number has increased over the last decade due to increasing maternal age and improved access to assisted reproductive technologies.^2^, ^3^ According to the latest Euro‐Peristat report, the incidence of multiple pregnancies in Italy has continued to rise, increasing from 15 per 1000 in 2010 to 16.4 per 1000 in 2015, with stability observed from 2015 to 2019. Unfortunately, the cesarean section (CS) rate in Italy remains among the highest in Europe, reaching 33% in 2019, compared to 26% across Europe overall.^4^ In twin pregnancies, the CS rate reported in Italy was 81.4%.^5^ Among the various challenges in managing twin pregnancies, the mode of delivery is one of the most crucial decisions to be made before birth.

Although obstetric practice has shifted towards cesarean for twins over the past several decades, evidence indicates that vaginal birth is preferable in most circumstances. Some studies have proposed that planned CS for twins may reduce the risk of adverse perinatal outcomes.^6^, ^7^ However, many other studies conclude that vaginal birth should be the standard of care, given that routine cesarean offers no benefit and adds risk, particularly long‐term risk to the mother and to her future pregnancies.^8^, ^9^, ^10^, ^11^ A recent systematic review and meta‐analysis concluded that planned vaginal birth and planned cesarean in twin pregnancies have comparable maternal and perinatal outcomes, supporting individualized decision making.^12^ Routine CS also offers no benefit for breech‐first twin pregnancies, which are often cited as the reason for CS.^13^, ^14^, ^15^, ^16^ Despite the recognized safety of vaginal twin birth, as shown in The Twin Birth Study,^8^ a randomized controlled trial of cephalic‐first twins; guidelines from the American College of Obstetricians and Gynecologists (ACOG)^17^; and the National Institute for Health and Care Excellence,^18^, ^19^ the rate of cesarean deliveries in twin gestations remains high.^20^, ^21^

But with diminishing experience due to high cesarean rates, how can clinicians gain the necessary skills that are necessary in emergency conditions such as incoming and unstoppable delivery? Numerous studies indicate that simulation‐based training is effective in improving technical skills, enhancing awareness of clinical situations, and improving event management, thus significantly reducing maternal and neonatal risks.^22^ Training in the management of twin birth, particularly for the extraction of the second twin, is crucial for all hospital centers that support vaginal birth in multiple pregnancies.^23^, ^24^ Simulation‐based training can help build greater confidence during the vaginal birth of the second twin, potentially leading to reduced maternal and neonatal complications.^25^, ^26^, ^27^, ^28^ It is widely recognized that having an experienced provider^29^ and implementing a systematic training program^30^ can lower the originally high elective cesarean rates.

To evaluate how effectively systematic training and specialist teams can impact twin CS rates, we studied the clinical outcomes and effects on provider attitudes and behavior in two hospitals. Additionally, we assessed the incidence of adverse maternal and neonatal outcomes in twin pregnancies before and after the intervention. Finally, we assessed providers' perspectives on whether the implementation improved their skill, knowledge, and confidence levels as well as their clinical practice during twin births.

## 2 MATERIALS AND METHODS

This was a retrospective multicenter study. The study period lasted 8 years, starting on January 1, 2017 and ending on December 31, 2024 in Santo Spirito Hospital in Pescara (Italy), and starting on January 1, 2019 and ending on December 31, 2024 in San Giovanni di Dio Hospital in Florence (Italy), with the mid‐point corresponding to organizational changes in the management of twin pregnancies in each hospital. On January 1, 2021 in Pescara, the obstetric team—consisting of senior physicians, resident doctors, and midwives—implemented quarterly training sessions focused on the management of twin delivery, breech delivery, shoulder dystocia, operative vaginal delivery, and other obstetric emergencies. This would typically consist of a lecture, then hands‐on practice on an obstetric mannequin for the remainder of the day. On July 1, 2022 in Florence, two teams skilled in twin and breech vaginal delivery were implemented, giving almost all women the opportunity of a twin vaginal birth, during both daytime and nighttime. We analyzed the impact of these interventions on the percentages of vaginal births, as well as the rates of both emergency and elective cesarean sections. The initial data collection using hospital discharge forms was conducted using the digital birth records of the obstetrics and neonatology units of each hospital. The data collection process was refined through additional searches and reviews of individual medical records. The data were then de‐identified. Physicians at both hospitals also completed qualitative surveys after the end of the study period assessing changes in their skill, knowledge, and confidence levels.

The inclusion criteria were as follows: patients over 18 years old; an uncomplicated diamniotic twin pregnancy (irrespective of chorionicity); gestational age of ≥34 weeks, with accurate pregnancy dating confirmed by ultrasound in the first trimester; and cephalic presentation of the first twin. Preterm births before 34^+0^ weeks' gestation, twin pregnancies with a non‐vertex presenting twin, intrauterine demise of one twin, monochorionic monoamniotic twins, triplets, and higher‐order multiples were excluded.

Pre‐intervention, CS was routine for most women, and few were offered the choice of mode of birth. Post‐intervention, patients were given the option to choose their mode of birth (planned vaginal birth or planned CS) after counseling with a senior physician, who explained the risks and benefits associated with both delivery methods, except in cases requiring emergency cesarean sections. Upon admission, an obstetric ultrasound was performed to evaluate fetal presentations, amniotic fluid volume, and the presence of cardiac activity in both fetuses. A cardiotocographic trace was obtained to assess fetal well‐being and uterine contractions, and an obstetric examination was performed to evaluate cervical status. Patients received informed consent documentation in the case of planned cesarean or labor induction if indicated. Indications for elective CS included the non‐cephalic presentation of the first twin and the patient's autonomy in decision making. Indications for labor induction included reaching 38 weeks for dichorionic twins, 36 weeks for monochorionic twins, or premature rupture of membranes.

The primary outcome was the mode of delivery: successful vaginal birth for both twins (either spontaneous or operative) versus cesarean deliveries (including combined vaginal/surgical births). Secondary outcomes included the incidence of adverse maternal and neonatal outcomes. Adverse maternal outcomes included postpartum hemorrhage (defined as an estimated blood loss of 1000 mL or more) and third‐ or fourth‐degree perineal tears. Adverse neonatal outcomes included an Apgar score <7 for one or both twins at 5 min and admission to neonatal intensive care.

After the second evaluation period, a Google form questionnaire was administered to labor ward specialists in the two hospitals. The questionnaire first gathered demographic data such as age, seniority, and previous experiences in twin vaginal birth during and after residency with both mannequins and actual patients. Next, participants evaluated their experiences before and after training, assessing whether the intervention had improved their skills, behaviors, attitudes, and confidence levels (see Supplementary File 1 for a list of survey questions).

Statistical analysis was conducted using IBM SPSS Statistics 23.0. Continuous variables are reported as means and standard deviations (SDs), while discrete variables are reported as numbers and percentages. Comparisons of continuous variables were analyzed using the *t*‐test, while discrete variables were compared using Pearson's Chi‐square and Fisher exact test as appropriate. Multivariate logistic regression analysis was employed to identify independent variables predicting binary outcomes. A two‐tailed *P* value of less than 0.05 was considered statistically significant.

In accordance with guidelines for retrospective observational studies, no ethical approval was required for this study, as it was a retrospective cohort evaluation of departmental management and outcomes.^31^, ^32^ The study was registered on www.ClinicalTrials.gov (NCT06628843).

## 3 RESULTS

**FIGURE 1:**
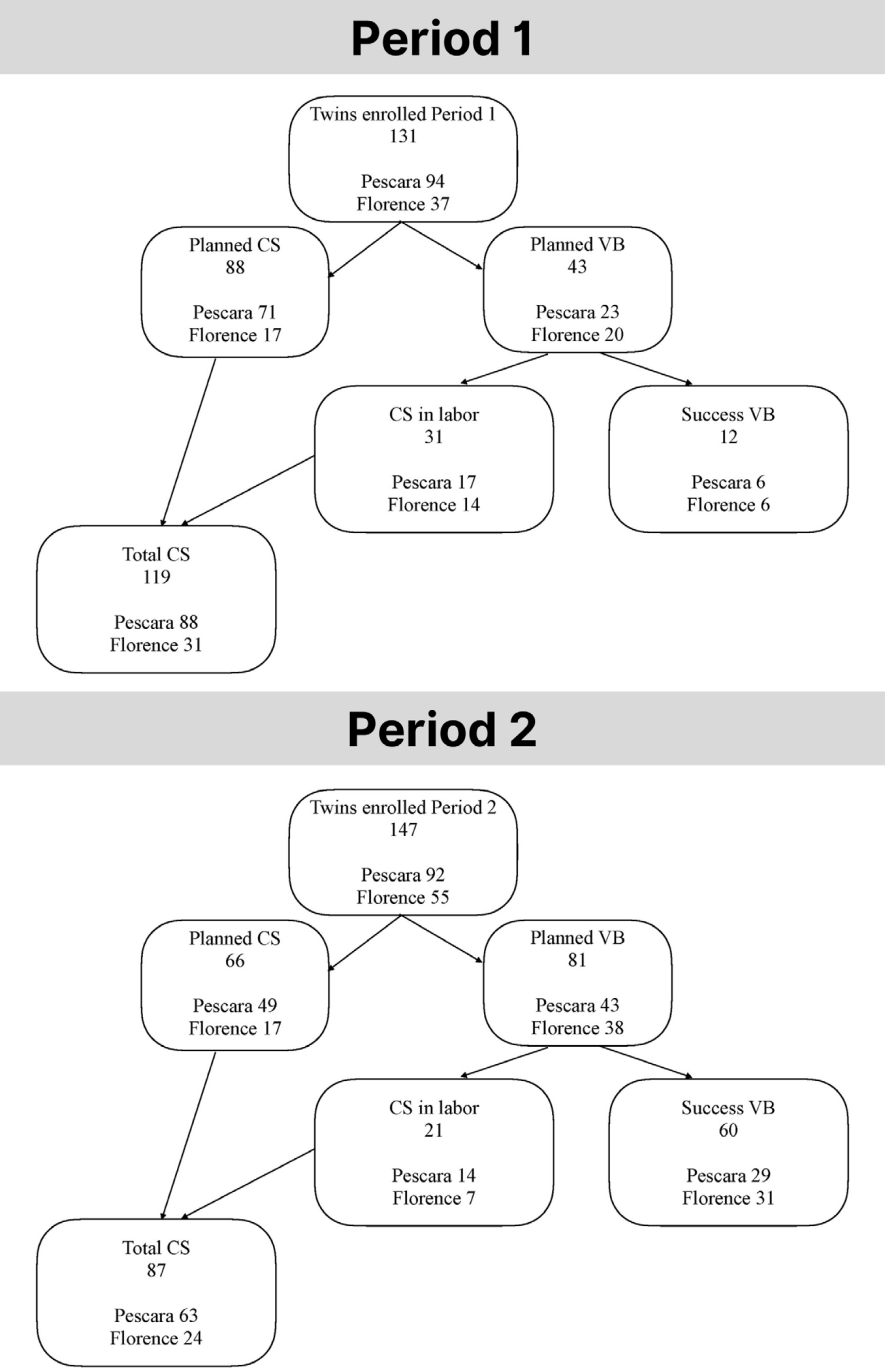
Mode of birth in Group 1 and 2 (pre‐ and post‐intervention).

During the study period the total number of deliveries in the two hospitals was 24 342. A total of 278 twin pregnancies of 34 weeks' gestation or more were eligible for analysis, for a total of 556 newborns. A total of 103 additional multiple pregnancies ≥34 weeks were excluded, including 91 with a non‐cephalic first twin, four triplet pregnancies, four monochorionic monoamniotic pregnancies, two pregnancies with uncertain chorionicity, and three patients whose medical records could not be evaluated. Out of the participants, 131 patients were in the pre‐intervention group, and 147 patients were in the post‐intervention group. Figure 1 shows the patient flow from enrollment to delivery in the two groups.

Data collection was stratified and analyzed in the two groups. Table 1 reports the population characteristics. Evaluating the association between clinical characteristics and CS rates overall and individually in Pescara and Florence, no statistically significant differences were found for any characteristics except for a slightly higher incidence of monochorionic pregnancies and slightly higher CS rates for those pregnancies (*P* < 0.001, *P* < 0.001, and *P* = 0.038, overall, Pescara, and Florence, respectively).

**TABLE 1:** Clinical characteristics.

	Period 1									Period 2								
	Total			Pescara			Florence			Total			Pescara			Florence		
	Total (131)	VB (12)	CS (119)	Total (94)	VB (6)	CS (88)	Total (37)	VB (6)	CS (31)	Total (147)	VB (60)	CS (87)	Total (92)	VB (29)	CS (63)	Total (55)	VB (31)	CS (24)
Maternal age	34.7 ± 6.3	37.2 ± 5.1	34.5 ± 5.3	35.3 ± 6.1	37.0 ± 3.6	35.2 ± 6.2	37.8 ± 7.8	35.0 ± 5.5	38.0 ± 8.1	35.3 ± 6.0	35.1 ± 6.4	35.0 ± 6.8	34.3 ± 5.6	32.5 ± 6.6	35.1 ± 5.0	33.8 ± 5.5	33.2 ± 6.4	34.8 ± 3.7
BMI	26.9 ± 5.3	27.3 ± 5.1	26.8 ± 5.3	26.5 ± 5.7	27.7 ± 6.3	26.4 ± 5.7	23.6 ± 3.6	22.0 ± 3.4	24.0 ± 3.7	26.2 ± 5.6	27.1 ± 5.5	24.8 ± 5.7	27.9 ± 4.6	26.9 ± 3.7	28.4 ± 5.0	28.8 ± 11.4	25.5 ± 3.3	35.5 ± 21.9
ART (%)	37 (28.2)	5 (41.7)	32 (26.8)	27 (28.7)	2 (33.3)	25 (28.4)	10 (27.0)	3 (50.0)	7 (22.6)	34 (23.1)	18 (30.0)	16 (18.4)	25 (27.2)	7 (24.1)	18 (28.6)	9 (16.4)	6 (29.0)	3 (12.5)
Nulliparous (%)	77 (58.9)	6 (50.0)	71 (59.7)	59 (62.7)	2 (33.3)	57 (64.8)	18 (48.6)	4 (66.7)	14 (45.2)	84 (57.1)	22 (36.7)	62 (71.3)	54 (58.7)	11 (37.9)	43 (68.2)	30 (54.5)	11 (35.5)	19 (79.2)
Previous CS (%)	21 (16.0)	0 (0.0)	21 (17.6)	17 (18.1)	0 (0.0)	17 (19.3)	4 (10.8)	0 (0.0)	4 (12.9)	17 (11.6)	4 (6.7)	13 (14.9)	10 (10.9)	2 (6.9)	8 (12.7)	7 (12.7)	2 (6.4)	5 (20.8)
MCDA (%)	17 (13.0)	2 (16.7)	15 (12.6)	13 (13.8)	1 (16.7)	12 (13.6)	4 (10.8)	0 (0.0)	4 (12.9)	23 (15.6)	11 (18.3)	12 (13.8)	14 (15.2)	1 (3.8)	13 (20.6)	9 (26.4)	5 (16.1)	4 (16.7)
Maternal diseases (%)	56 (42.7)	6 (50.0)	50 (42.0)	47 (50.0)	4 (66.7)	43 (48.8)	9 (24.3)	2 (33.3)	7 (22.6)	57 (38.8)	16 (26.7)	41 (47.1)	49 (53.3)	13 (44.8)	36 (57.1)	8 (14.5)	3 (9.7)	5 (20.8)
Fetal diseases (%)	33 (25.2)	0 (0.0)	33 (27.7)	25 (26.6)	0 (0.0)	25 (28.4)	8 (21.6)	0 (0.0)	8 (25.8)	32 (21.7)	7 (11.7)	25 (28.7)	24 (26.1)	2 (6.9)	22 (34.9)	8 (14.5)	5 (16.1)	3 (12.5)
Gestational age (weeks)	35.2 ± 12.7	37.5 ± 0.7	35.0 ± 1.4	35.5 ± 6.1	37.0 ± 0.9	36.0 ± 2.8	36.8 ± 8.0	35.8 ± 1.5	36.3 ± 1.1	35.3 ± 6.0	36.2 ± 1.3	36.3 ± 1.3	34.3 ± 5.6	35.5 ± 2.1	36.0 ± 2.7	33.8 ± 5.5	35.9 ± 1.1	36.4 ± 1.2

*Note*: BMI, calculated as weight in kilograms divided by the square of height in meters.

Abbreviations: ART, assisted reproductive technologies; BMI, body mass index; CS, cesarean section; MCDA, mono‐chorionic di‐amniotic; VB, vaginal birth.

Table 2 describes birth outcomes. The incidence of vaginal birth increased significantly in the second period, whether considering both hospitals together or each hospital individually (*P* < 0.0001, *P* < 0.0001, *P* = 0.0001; overall, Pescara, and Florence respectively). The incidence of nulliparity versus multiparity was not significantly different between the two study periods (*P* = 0.0004, *P* = 0.0161, and *P* = 0.008, respectively). No statistically significant differences were found for maternal and neonatal adverse outcomes analyzed between the before and after groups (see Table 2), although this study is underpowered to detect differences in rare events such as neonatal death or severe neonatal morbidity. Apgar scores of <7 for the first and/or the second twins were comparable between the two groups. The number of neonatal intensive care unit admissions was higher for newborns delivered by CS in both groups (17 vs. 0, and 25 vs. 11; CS vs. vaginal delivery), but this difference was not statistically significant (*P* = 0.899).

**TABLE 2:** Mode of birth and maternal and neonatal outcomes.

	Period 1									Period 2									*P* value
	Total			Pescara			Florence			Total			Pescara			Florence			
	Total (131)	VB (12)	CS (119)	Total (94)	VB (6)	CS (88)	Total (37)	VB (6)	CS (31)	Total (147)	VB (60)	CS (87)	Total (92)	VB (29)	CS (63)	Total (55)	VB (31)	CS (24)	
VB rate (%)	9.2%			6.4%			15.8%			40.8%			31.5%			56.4%			0.0001
Planned VB	43 (32.8%)			23 (24.5%)			20 (54.1%)			81 (55.1%)			43 (46.7%)			38 (69.1%)			0.0221
Planned CS (%)	88 (67.2%)			71 (75.5%)			17 (45.9%)			66 (44.9%)			49 (53.3%)			17 (30.9%)			0.0301
Cesarean in labor (%)	31 (23.7%)			17 (19.3%)			14 (22.6%)			21 (24.1%)			14 (22.2%)			7 (12.7%)			NS
5‐min APGAR <7^a^ (%)		0 (0.0%)	1 (0.8%)		0 (0.0%)	1 (1.1%)		0 (0.0%)	0 (0.0%)		0 (0.0%)	0 (0.0%)		0 (0.0%)	0 (0.0%)		0 (0.0%)	0 (0.0%)	NS
Adv. maternal outcome^b^ (%)		2 (16.7%)	5 (4.2%)		0 (0.0%)	0 (0.0%)		2 (33.3%)	5 (16.1%)		8 (13.3%)	9 (10.3%)		1 (3.4%)	6 (9.5%)		7 (22.6%)	3 (12.5%)	NS
Adv. neonatal outocome^c^ (%)		0 (0.0%)	17 (14.3%)		0 (0.0%)	12 (13.6%)		0 (0.0%)	5 (16.1%)		11 (18.3%)	25 (28.7%)		5 (17.2%)	23 (36.5%)		6 (19.3%)	2 (8.3%)	NS

Abbreviations: CS, cesarean section; NICU, neonatal intensive care unit; NS, not significant; VB, vaginal birth.

^a^ Occurring in twin A and/or B.

^b^ PPH >1000 mL or third/fourth degree tear.

^c^ Defined as NICU admission.

**FIGURE 2:**
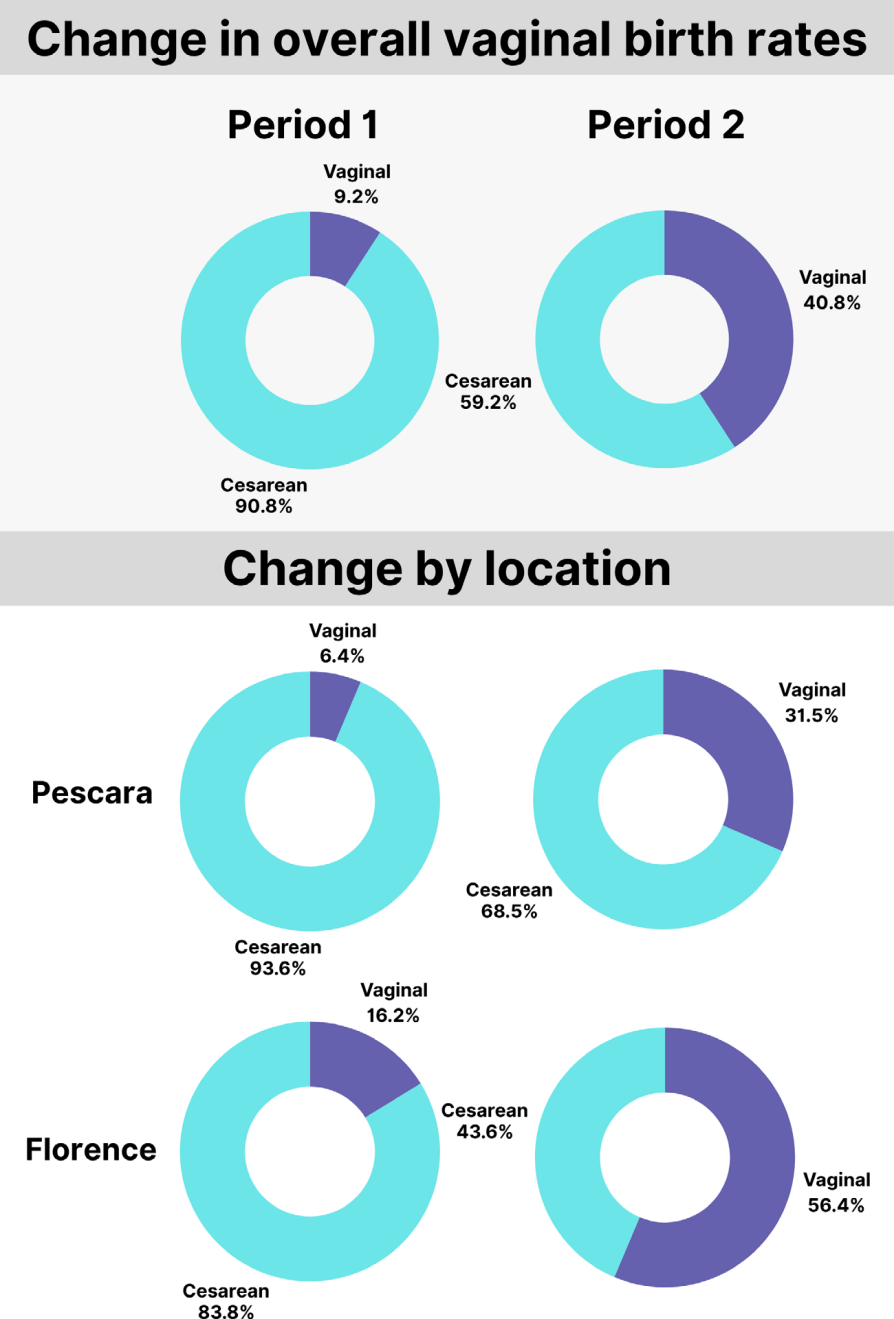
Overall change in vaginal twin birth rates pre‐ and post‐implementation by location.

Figure 2 shows the changes in vaginal birth rates pre‐ and post‐intervention, increasing from 9.2% pre to 40.8% post overall, and variations in vaginal twin birth rate in each site. In Pescara, it increased from 6.4% to 31.5%, and in Florence it increased from 16.2% to 56.4%.

**FIGURE 3:**
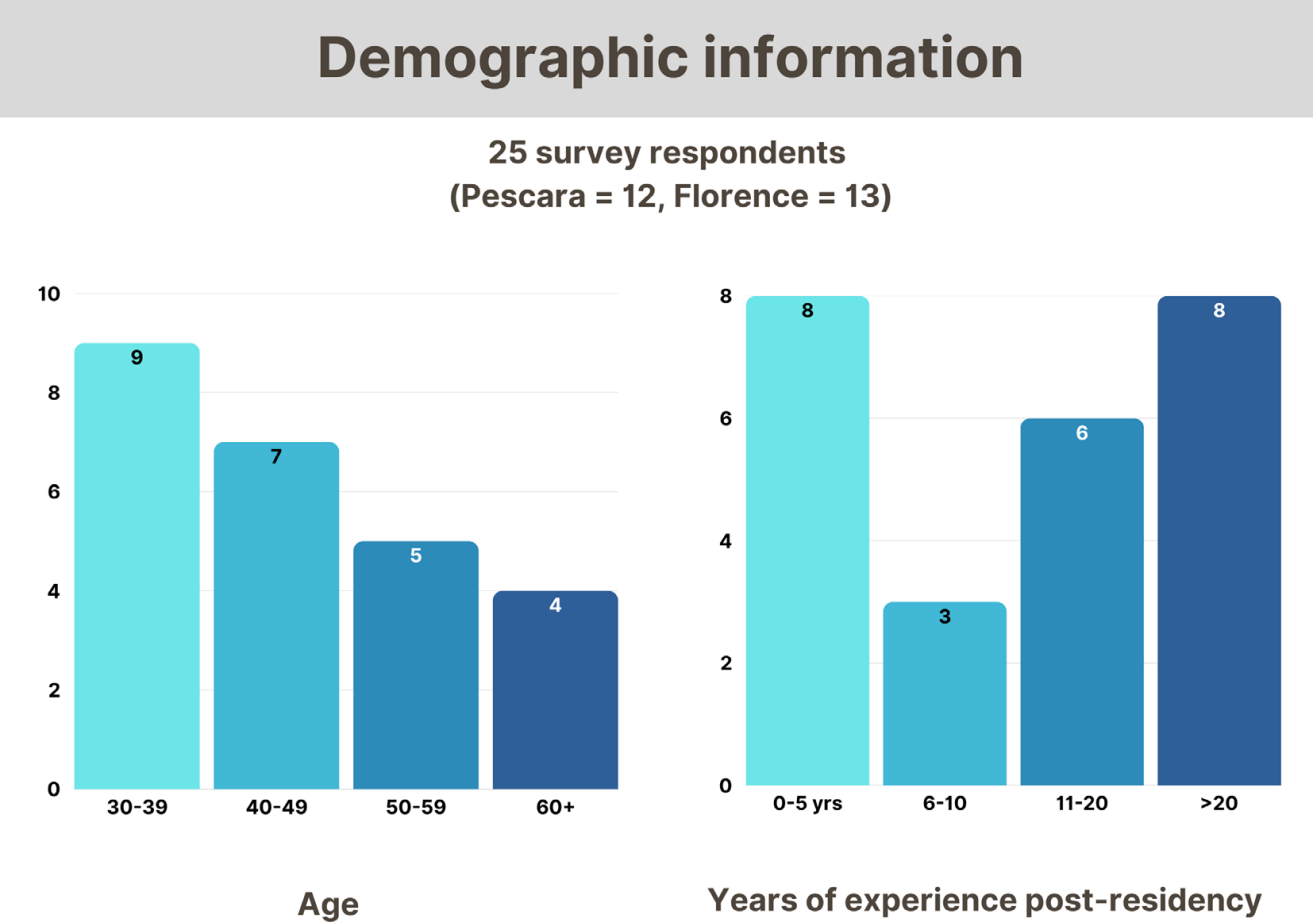
Provider demographic information (age and experience levels).

**FIGURE 4:**
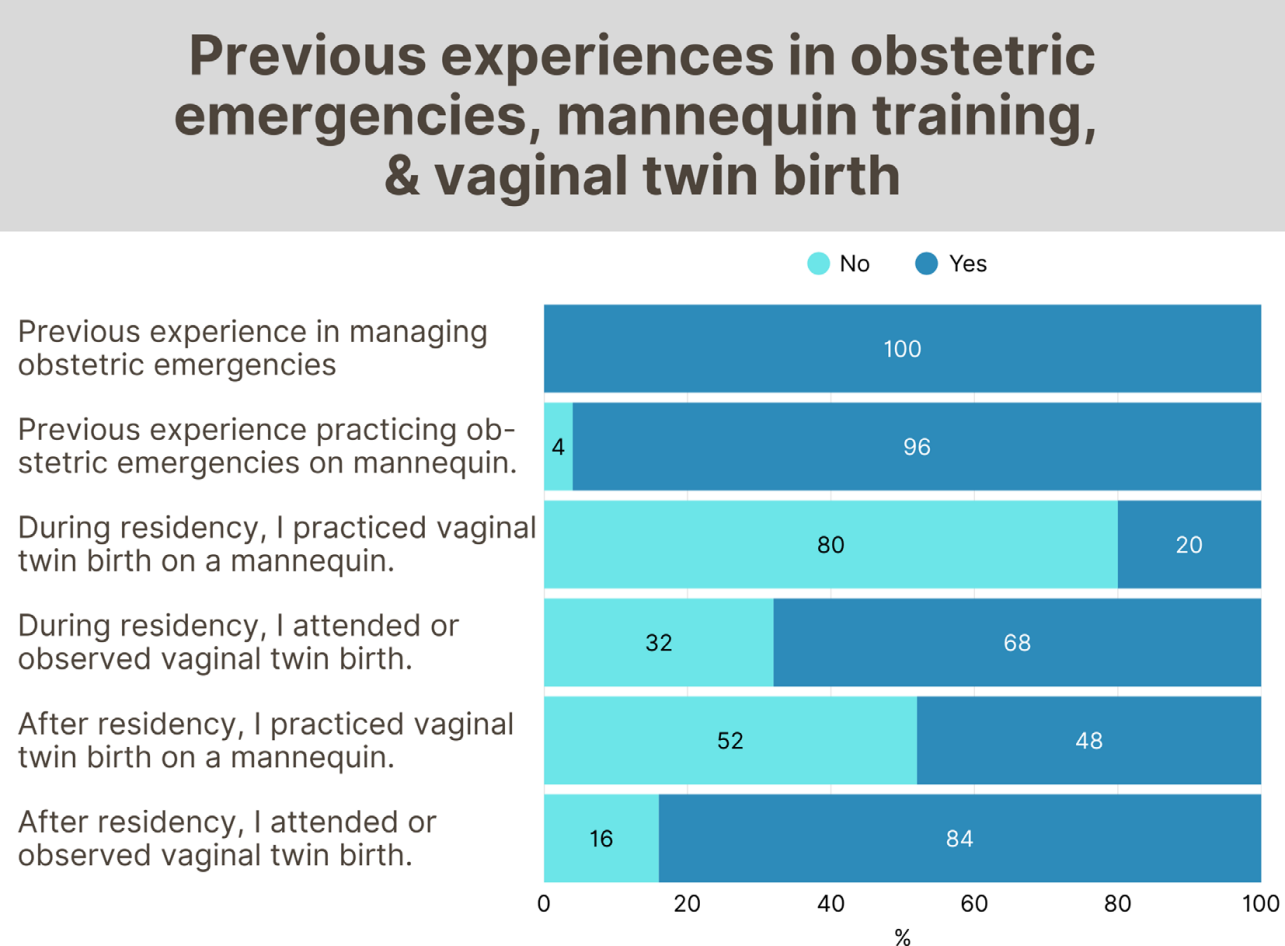
Previous experiences in obstetric emergencies, mannequin training, and vaginal twin birth.

Out of 28 specialists working regularly in the two hospitals, 25 completed the questionnaire (89% response rate). The rate of specialists attending vaginal twin births in the pre‐intervention period was similar in both hospitals (7 out of 12 in Pescara, 7 out of 13 in Florence, *P* = 0.821), while the number of specialists during the post‐intervention period increased to 12 out of 12 in Pescara and 12 out of 13 in Florence (*P* = 1.000). The questionnaires revealed consistent agreement about the value and impact of the training programs for improving behavior, confidence levels, awareness, and skill levels. Figures 3 and 4 show the demographic results and levels of previous experience in obstetric emergencies and vaginal twin birth. Attendees had slightly more experience with simulated or real vaginal twin birth after than during residency, but high levels of training in managing obstetric emergencies both during and after residency.

**FIGURE 5:**
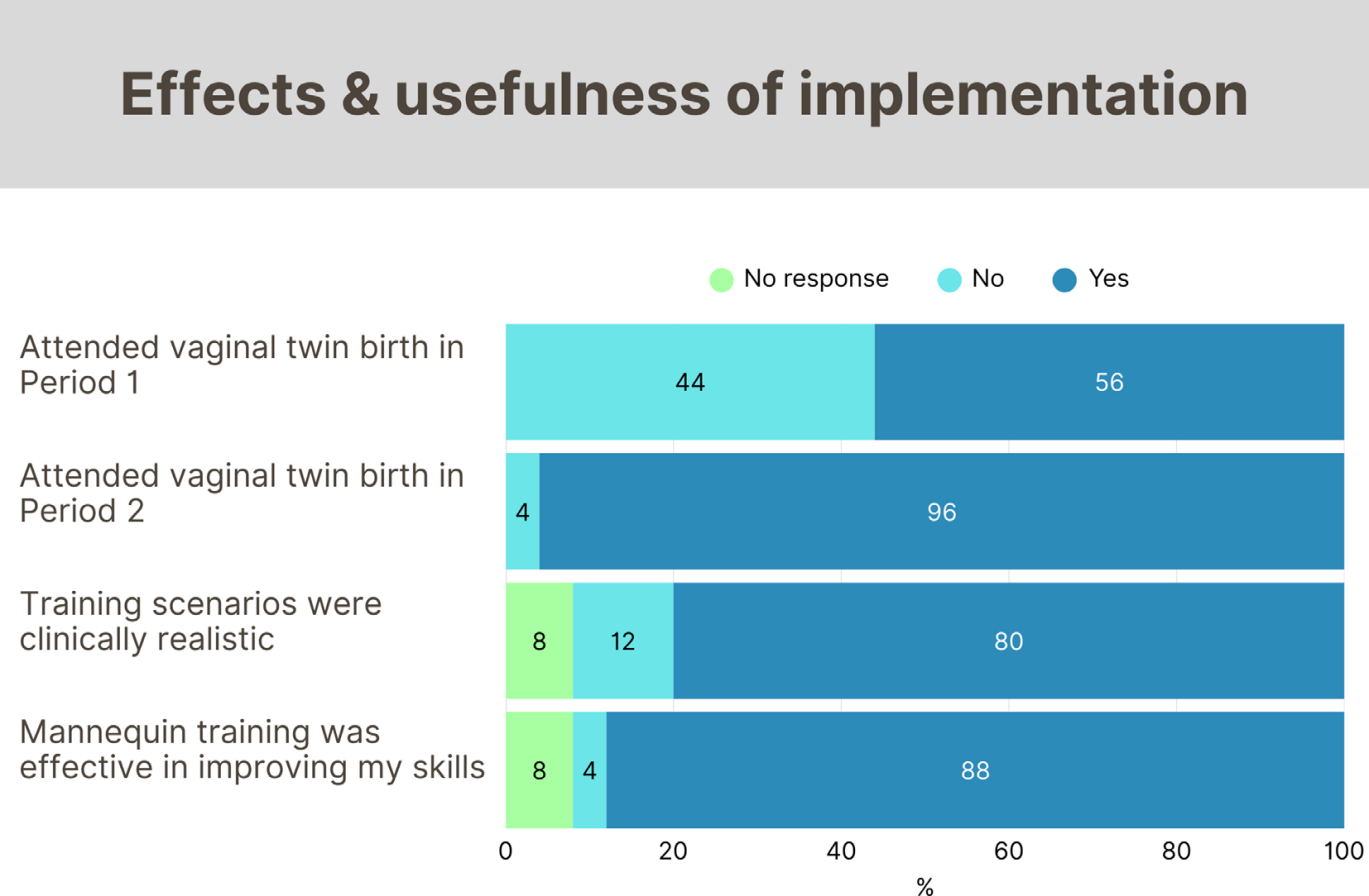
Effects and usefulness of implementation.

**FIGURE 6:**
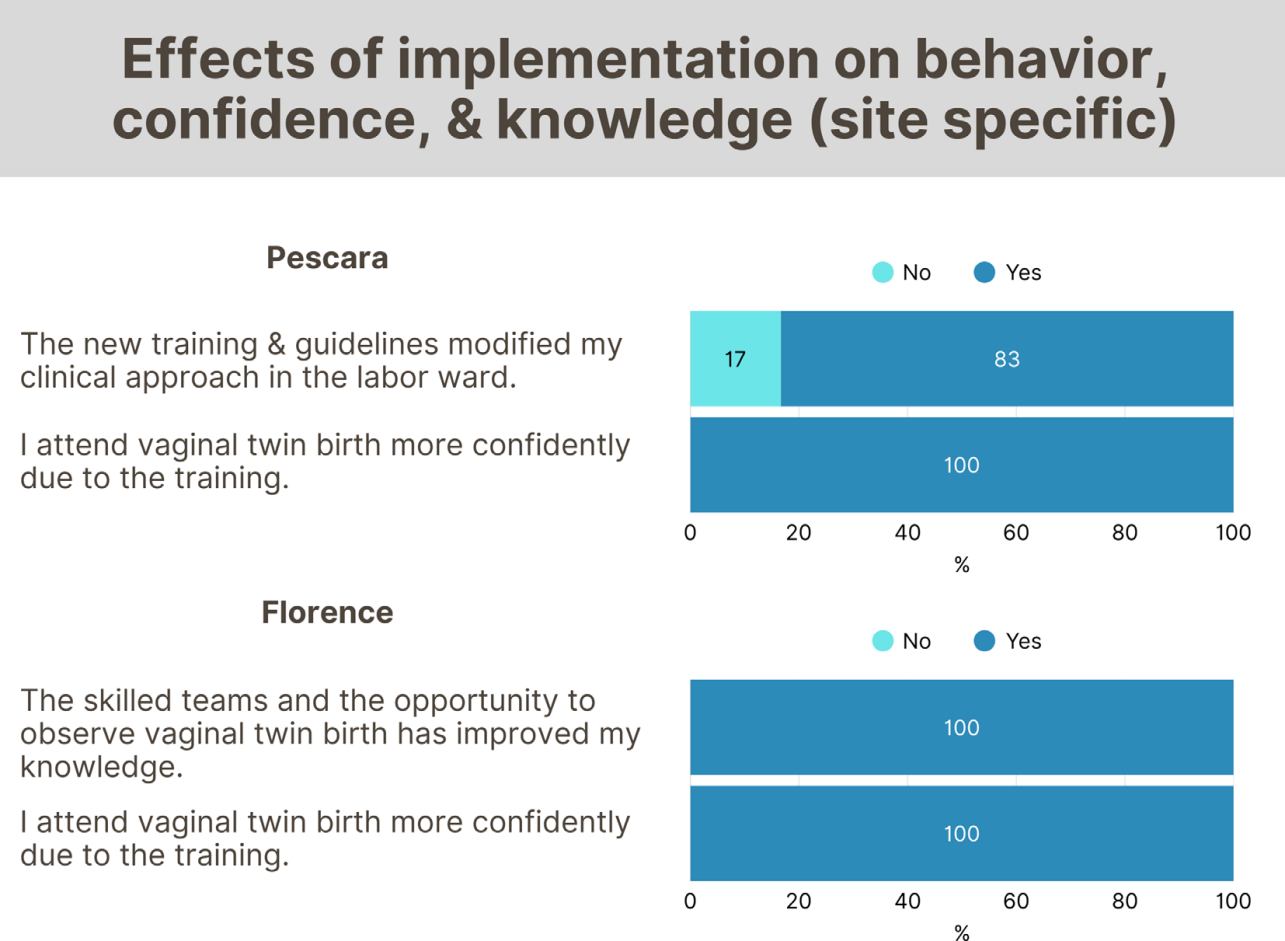
Effects of implementation on behavior, confidence, and knowledge levels (site specific).

**FIGURE 7:**
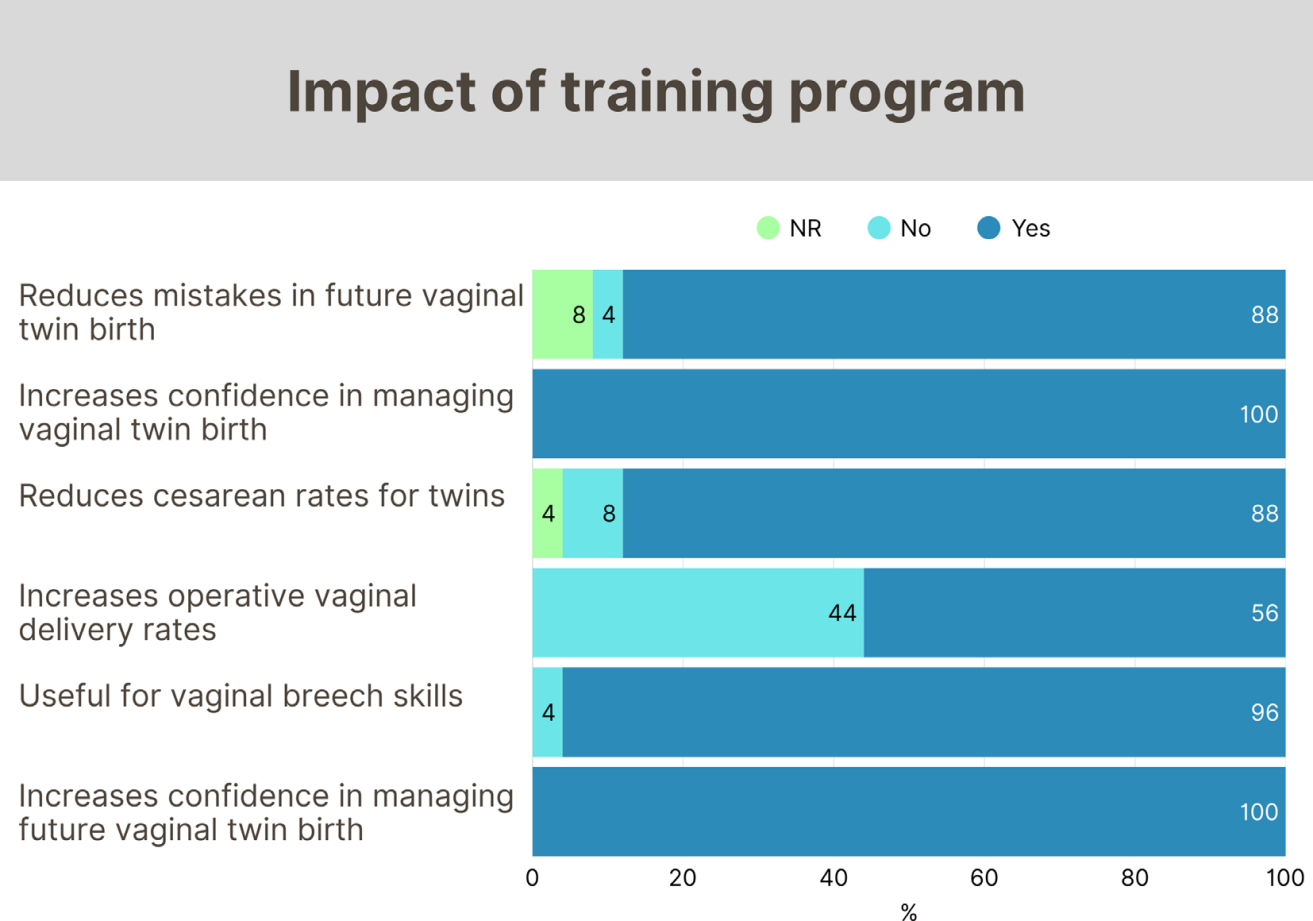
Impact of the intervention on knowledge, skill, and clinical practice.

Figures 5, 6, 7 show the results of the training intervention, both site‐specific and overall. Confidence levels increased identically in both locations. Except for the effect on operative vaginal delivery rates, participants strongly agreed that the intervention reduces future mistakes, increases knowledge, lowers CS rates, improves vaginal breech birth skills, and improves future management of vaginal twin birth.

## 4 DISCUSSION

Our study demonstrated that, in settings with a high background CS rate for twin pregnancies, two different interventions (periodic training for healthcare staff and availability of teams skilled in twin and breech vaginal birth) proved effective in increasing vaginal twin birth rates. Population characteristics did not show significant differences in the pre‐ and post‐intervention groups, with the only exception of a higher incidence of nulliparity within vaginal births in the post‐intervention groups. Following the introduction of periodic training for healthcare staff, the incidence of vaginal twin births at Pescara Hospital significantly increased starting in 2021, rising from 6% before 2021 to 32% afterwards. This change resulted in a significant reduction of the CS rate for twin pregnancies where no other obstetric indications warranted a CS. In Florence, with the introduction of new specialized teams actively involved in attending vaginal twin births, the vaginal birth rate rose from 16% to 56%.

This evidence highlights the importance of continuous training for healthcare personnel, as supported by existing literature,^25^, ^26^, ^27^, ^33^, ^34^, ^35^, ^36^, ^37^ which confirms that dedicated training programs can enhance maternal‐fetal outcomes by reducing the incidence of unnecessary cesarean sections. Our study shows changes consistent with Kirkpatrick Level 2 (learning) in the providers' improved knowledge, skill, and confidence level. More significantly, the achievement of measurable results (reduction in CS with no worsening of maternal or neonatal outcomes) is consistent with Kirkpatrick Level 4 (results).^38^

The observed improvement is likely attributed to increased awareness and confidence among healthcare staff in managing twin labors due to the training they received in Pescara, and even more in Florence due to adding teams skilled in and dedicated to supporting vaginal twin birth. When analyzing adverse outcomes such as postpartum hemorrhage (>1000 cc), severe perineal trauma, 5‐min Apgar scores <7, and the number of infants admitted to the neonatal intensive care unit, no statistically significant differences were found between the two periods.

The two hospitals in this study had different interventions that resulted in different increases in vaginal twin birth rates. In Pescara, the strategy was to re‐train the entire labor ward via periodic skills training. In Florence, the strategy was to create two skilled specialist teams but not to train the entire staff.^30^ Our results suggest that dedicated teams may be able to support a more rapid implementation of vaginal twin birth, as evidenced by the higher vaginal birth rates in Florence. However, it is possible that with more time, the vaginal birth rate in Pescara may also be able to attain similar figures. We anticipate that with additional training and more years of data collection, the rate of vaginal twin birth could continue to rise to levels similar to or higher than those found in the JUMODA study,^10^ with a 61% overall vaginal birth rate for cephalic‐first twins and 80.3% of planned vaginal twins ending in successful vaginal births. Reskilling is not an immediate process, but it is imperative to train maternity care providers in vaginal breech and twin birth to ensure that women can choose their mode of birth and avoid the risks of CS. Specialist teams supporting vaginal twin birth could be a short‐term strategy with department‐level skills training as a long‐term strategy.

The increase in CS rate is a difficult trend to reverse. As the authors of a study on vaginal twin birth noted, “scientific evidence and society opinion are likely insufficient to reverse the national trends that favor cesarean delivery for twins. Instead, implementation of provider training and support programs is critical for increasing the rates of twin vaginal birth.”^38^ Our iniatitives demonstrate two strategies to increase the rate of vaginal twin birth without compromising maternal or neonatal outcomes, both provider‐focused. Our findings are different from a similar study,^39^ which also compared outcomes before and after an initative to increase the rate of vaginal twin births. While their vaginal birth rates increased post‐initative, the increase was much less dramatic and mostly attributed to secular change.

Emerging research also demonstrates novel strategies to increase the fidelity of simulation training, such as using water‐filled balloons with a fetal doll inside to mimic the feel of performing breech extractions with an intact amniotic sac,^23^, ^40^, ^41^ adapting simulators into upright birth positions,^42^ and developing more realistic soft tissues.^43^ We encourage further innovation to help simulation training approach the realism of attending live births.

The staff experience with this intervention was very positive, with almost everyone feeling that the implementation improved vaginal birth skills, knowledge, and confidence levels and decreased CS rates. This study is significant because it also tracks clinical outcomes post‐training, something that many studies on simulation training lack. Our study shows a correlation between training and actual changes in clinical practice (higher vaginal birth rate) as well as no worsening of maternal or neonatal outcomes.

These results may not be replicable in hospitals where the staff are unsupportive of vaginal twin birth or where specialists are unable or unwilling to form supportive teams. We acknowledge that in some parts of the world, vaginal twin/breech birth is met with resistance and sometimes outright bans, due to medico‐legal concerns or cultural beliefs in CS as a safer option. Our results are also underpowered to detect rates of rare adverse outcomes. It is also unknown whether in the long‐term the vaginal birth rate will continue to rise, to plateau, or to fall after the implementation of vaginal twin intervention. Our study examined only twin pregnancies with the first baby in a vertex position. The same results may be possible when the first baby is presenting breech, but this was beyond the scope of this project. Because the literature shows that vaginal birth of twins with the first baby in a breech position is also a reasonable option,^44^ we encourage other centers with teams skilled in both vaginal breech and twin birth to publish their outcomes for breech‐first as well as head‐first twins.

## 5 CONCLUSION

From this study, we can conclude that a rapid increase in the vaginal birth rate of twins is possible via either staff‐wide training or specialist teams without adversely affecting maternal or neonatal outcomes. Provider knowledge, skill, and confidence levels also increase after periodic training.

## AUTHOR CONTRIBUTIONS

C.C., C.M., F. P., B.M., M.M. and M.R., planned the study. C.C. and R.F., interpreted the results. C.C., M.M. and F.C., collected data from Pescara. C.M., C.M. and A.P., collected data from Florence. R.F. and C.C., analyzed the final data set. C.C., B.M., M.M., C.M. and R.F., wrote the first draft of the manuscript. R.F., C.C. and F.P., critically reviewed the manuscript.

## FUNDING INFORMATION

The authors received no funding for this study.

## CONFLICT OF INTEREST STATEMENT

The authors confirm there are no conflicts of interest.

## Supporting information


**File S1:** Pescara & Florence Provider Survey Questions (Translated from Italian).

**Data S1:** STROBE‐checklist.

**Data S2:** Supplementary Information.

## Data Availability

The data that support the findings of this study are available as supplementary files.
