# Supplementary material for: Evaluating the effect of two different training interventions on improving vaginal twin birth rates and provider confidence and knowledge levels: A pre‐ and post‐intervention study
Source: Int J Gynaecol Obstet. 2026 Mar 7;174(2):1025–35. doi: 10.1002/ijgo.70947 (PMC13377334; doi:10.1002/ijgo.70947)
Supplement: Supplementary file 1 — File S1: Pescara & Florence Provider Survey Questions (Translated from Italian). [file IJGO-174-1025-s001.docx]

**Pescara & Florence Provider Survey Questions**

**(Translated from Italian)**

Email : _________________________________

| Age | 30-39 | 40-49 | 50-59 | 60+ |
| --- | --- | --- | --- | --- |
| Years of experience post-residency | 0-5 | 6-10 | 11-20 | 20+ |
| Hospital | Pescara | | Florence | |
| During and/or after my residency, I have had previous experience in managing obstetric emergencies. | | | Yes | No |
| During and/or after my residency, I have had the opportunity to practice obstetric emergencies on a mannequin. | | | Yes | No |
| During my residency I had the opportunity to practice​​ vaginal twin birth on a mannequin. | | | Yes | No |
| During my residency, I had the opportunity to attend or observe vaginal twin births. | | | Yes | No |
| After residency, I have had the opportunity to practice vaginal twin birth on a mannequin. | | | Yes | No |
| After residency, I have had the opportunity to attend or observe vaginal twin births. | | | Yes | No |
| In period 1, I attended vaginal twin births. | | | Yes | No |
| In period 2, I attended vaginal twin births. | | | Yes | No |
| Were the training scenarios you participated in realistic in respect to clinical practice? | | | Yes | No |
| Was the mannequin training effective in improving your skills? | | | Yes | No |
| (Pescara) The post-implementation labor word management and guidelines changed my clinical approach in the labor ward. | | | Yes | No |
| (Pescara) I attend vaginal twin birth more confidently due to the training. | | | Yes | No |
| (Florence) Has the introduction of skilled teams and the opportunity to observe vaginal twin births improved your skills in the labor ward? | | | Yes | No |
| (Florence) I attend vaginal twin births more confidently due to the training. | | | Yes | No |
| In your opinion, is the training helpful in reducing mistakes you made in the past during vaginal twin births? | | | Yes | No |
| In your opinion, has the training given you more confidence in managing vaginal twin births? | | | Yes | No |
| In your opinion, has the training reduced the number of cesarean sections for twins? | | | Yes | No |
| In your opinion, has the training increased the rate of operative vaginal deliveries? | | | Yes | No |
| Has the training in vaginal twin births also been useful for vaginal breech births? | | | Yes | No |
| Will you manage in the future twin vaginal birth with more confidence? | | | Yes | No |
