# Supplementary material for: Evaluating the effect of two different training interventions on improving vaginal twin birth rates and provider confidence and knowledge levels: A pre‐ and post‐intervention study
Source: Int J Gynaecol Obstet. 2026 Mar 7;174(2):1025–35. doi: 10.1002/ijgo.70947 (PMC13377334; doi:10.1002/ijgo.70947)
Supplement: Supplementary file 3 — Data S2: Supplementary Information. [file IJGO-174-1025-s003.pdf]

Da: BJOG: An International Journal of Obstetrics & Gynaecology onbehalfof@manuscriptcentral.com
Oggetto: BJOG: An International Journal of Obstetrics & Gynaecology - Decision on Manuscript ID BJOG-25-0982
Data: 22 ottobre 2025 alle ore 11:50
A: celentanoclaudio70@gmail.com

Dear Dr Celentano,

Thank you very much for submitting your manuscript "Reduction of planned cesarean delivery in twin pregnancy after a training intervention." to BJOG: An International Journal of Obstetrics & Gynaecology.

The referees and editors have considered your paper carefully, and unfortunately we have come to the conclusion that your paper cannot be accepted for publication in BJOG.

Your paper was considered by two reviewers and several editors, including a Deputy Editor-in-Chief. Please find the reviewers' comments and the editors' feedback below.

Sometimes reviewers' comments are positive and yet we still decide not to publish. We receive more submissions than we have space for; therefore we take into account relevance to our international readership, originality and appropriateness in relation to other papers in the Journal. Our decision does not necessarily imply a judgement of poor scientific quality.

However, your paper is a candidate for transfer to another journal published by Wiley or its affiliates. Please consider the journal options below and if you would like us to transfer your manuscript and associated reviews, click the transfer link. You will then be taken to the transfer portal site where you can select your preferred journal and initiate the transfer.

Reproductive, Female and Child Health:
* Open Access
* Aims to improve clinical practice in reproductive, female, & child health
* Research articles, reviews, case reports, clinical observations & commentaries
* Free format submission: https://authorservices.wiley.com/author-resources/Journal-Authors/Prepare/free-format-submission.html
* More at https://onlinelibrary.wiley.com/journal/27687228

To automatically transfer your paper and any related reviews, click the link below:

*** PLEASE NOTE: This is a two-step process. After clicking on the link, you will be directed to a webpage to confirm. ***

https://mc.manuscriptcentral.com/bjog?URL_MASK=1eaedd0191874acd899c322bbe24fde3

Pregnancy:
- OA journal
- Peer-reviewed original research papers, reviews, opinions, and guidelines devoted to the topic of pregnancy. The journal aims to include work that encompasses all aspects of pregnancy-related investigation, including those concerning the life course of the individual and social drivers of health.*
- Accepts Original Research Papers, Opinion Papers, Reviews, Brief Reports, Letters to the Editor (with approval from Editorial Office), Editorials (invitation only).
- Free Format submission: no manuscript formatting required.
- More: https://onlinelibrary.wiley.com/journal/29979684

To automatically transfer your paper and any related reviews, click the link below:

https://forms.office.com/Pages/ResponsePage.aspx?id=TyT-JA6J70a-L6UgKXa3pZ29tUuC-d5JoohbqMz3xlpUQTJFQ1hKOE5ZQjVCWjBNUUFGS0tGVzBLSCQlQCN0PWcu

Obstetrics and Gynecology International:
- OA journal
- Peer-reviewed research and review articles related to obstetrics, maternal-fetal medicine, gynecologic oncology, uro-gynecology, reproductive medicine, infertility, reproductive endocrinology, and sexual medicine.
- Free Format submission: no manuscript formatting required.
- More: https://onlinelibrary.wiley.com/journal/8272

To automatically transfer your paper and any related reviews, click the link below:

https://forms.office.com/Pages/ResponsePage.aspx?id=TyT-JA6J70a-L6UgKXa3peOQjQniSSRMrakBbiIqDc1UOTdZWVVITU9RRTBOWFZWN1E3VkE1SVVOSS4u

Please note that acceptance of the offer to transfer does not guarantee acceptance of your manuscript as the Editors of your selected journal will still need to undertake due evaluation of the manuscript. Once the transfer of the files, data and reviews is complete you will receive an email request asking you to log in to the submission site's Author Center, provide any missing information unique to that journal's submission process, and complete the submission.

If you are interested in transferring to a gold open access journal, or selecting an open access option in a subscription hybrid journal, visit http://bit.ly/WileyOAFunding to determine what funding is available. Institutions, countries, and other factors may result in partial or full coverage of the Article Publication Charge (APC).

Thank you for thinking of BJOG when considering publication. We will look forward to contributions from you and your colleagues in the future.

Sincerely,

Roxanne Hastie
Scientific Editor, BJOG: An International Journal of Obstetrics & Gynaecology

Reviewer(s)' Comments to Author:

Reviewer: 1

Comments to the Author

Overall, I really like the concept of the article, and the results are very promising—the dramatic decrease in cesarean sections is particularly noteworthy and could be emphasized more.

However, several areas could be improved:

The tables and figures are difficult to interpret and perhaps too numerous. The mode of delivery pre- and post-intervention could be presented more clearly and concisely.

Consider explicitly mentioning the Kirkpatrick levels; this study likely corresponds to level 4, the highest level, which should be highlighted.

The abstract should focus more on the high, non-evidence-based rates of cesarean sections for twins in Italy pre-intervention and the need to reduce them. The setting is mentioned twice, which makes the abstract somewhat unclear. The survey data could be removed to improve clarity.

Statements regarding “no effect on maternal and neonatal outcomes” are underpowered and should be interpreted cautiously.

The study combines three aspects—two hospitals and healthcare provider experiences. While interesting, this makes the narrative difficult to follow. Consider removing survey data for clarity.

Long quotes in the interpretation section, especially at the beginning, are not recommended.

Sentences such as “The number of neonatal intensive care unit admissions was higher for newborns delivered by cesarean section in both groups… but this difference was not statistically significant (p = 0.899)” could be simplified to: “No difference was observed.”

Many outcomes are likely underpowered; this should be clearly emphasized in the discussion.

The conclusion should summarize the study’s findings only, without introducing new suggestions or literature references.

Reviewer: 2

Comments to the Author
Thanks for allowing me to review your interesting manuscript. I have several comments and questions.

1. You make a very definite statement that "In accordance with guidelines for retrospective observational studies, no ethical approval was required for this study, as it is a retrospective cohort evaluation of departmental management and outcomes [30-31]. The study was registered on www.ClinicalTrials.gov (NCT06628843)." Can I ask if this was the advice received from your local Human Research Ethics Committee or if this is assumed to be the case? I am asking this because, although this is clearly based on analysis of retrospective clinical data, the whole purpose of the study is to examine an intervention (training and the provision of dedicated specialist teams), and that is what should interest an Ethics Committee. Can you please respond to this concern? In my own country a study such as this would require Ethics approval (which I am sure would be given).

2. In your manuscript you quite correctly say that it is not really possible to know if it is the education and training that made such a difference, or that it was the provision of dedicated specialist teams to manage the twin births. Or, of course, it could be both of these interventions working together. However, there is another possibility.

At the outset you state that women were given the choice of having a planned c/section or a planned vaginal birth. But was this exactly the same choice that they had before the intervention was planned? If the assumption is that most twins would be by c/section it would not be surprising if most women also chose to do that. Can you comment on if this could have influenced the results of your study? Is it simply reflecting that maternal choice is the most important factor?

3. I found Figures 1 & 2 quite confusing and they really show the same data as can be found in Figures 3 & 4. I had a couple of suggestions. Firstly,, showing the percentages in Figures 1 & 2 as well as the raw numbers would be an improvement, or the other option is to put the data underpinning Figures 1 & 2 in a table. Or else you could leave Figures 1 & 2 and the data contained out of the results completely, and simply present Figures 3 & 4.

4. Although the Twin Birth study (8) is briefly mentioned in your introduction this is one of the landmark papers about twin births, and a more comprehensive description and critique of the impact of that publication would be appropriate.

5. The perinatal outcomes in your study are very good, but how big a trial would be needed to show any change to the rare intrapartum outcomes such as stillbirth and/or perinatal asphyxia. Have you done sample size calculation for these outcomes?
